# The need for transparency in the treatment of spinal diseases

**DOI:** 10.1590/S1679-45082013000100002

**Published:** 2013

**Affiliations:** 1 Hospital Israelita Albert Einstein, São Paulo, SP, Brazil

The article entitled “*Spine surgery cost reduction at a specialized treatment center*,”^(1)^ and published in this edition of **einstein**, shows the economy of a specialized treatment center that performs a second-opinion service. In addition to findings of economic features, another factor that has drawn the reader's attention to this article are the differences in opinions concerning therapeutic management, highlighted by a second-opinion algorithm started after initial spinal surgery has been suggested. The conservative bias of this algorithm was reduced because patients could refuse to participate in the study, and there was the possibility of patients undergoing surgery with the surgeon who first confirmed the surgical indication. In fact, roughly 75% of patients in whom surgery was indicated could be treated without surgery.

The lack of evidence-based treatment guidelines for spinal disease is a reality. A systematic review carried out by Cheng et al. emphasized the need to develop guidelines for treatment of spinal diseases with methodologic quality, transparency, and in agreement with the Appraisal of Guidelines Research and Evaluation instrument^(2)^. This lack of guidelines to support the diagnosis and treatment of spinal disorders contributes to the wide variability of therapeutic management.

Another fact that negatively affects the development of guidelines is the speed in which new technologies are launched by the surgical device and implant industry, which is higher than the number of investigations conducted to prove their efficacy. The use of bone morphogenetic proteins (BMPs) in spinal fusion surgery is a good example of lack of data. Cahill et al., using the Nationwide Inpatient Sample database, showed that BMPs in spinal fusion yielded higher complications than procedures performed without BMPs^(3)^. Nowadays, the use of BMPs is almost prohibited in cases of cervical spinal fusion and has few discussible indications for lumbar spinal procedures. It is highly important to consider new medical devices carefully, even when results about such devices are published in the literature. A study conducted by Okike et al. showed that positive results often have conflicts of interest between authors and the implant industry^(4)^.

It is possible that conflicts of interest between surgeons and implant manufacturers should also be considered. Healy and Peterson, with the the US Department of Justice, investigated the relationship between the orthopedic device industry and orthopedists^(5)^. They reported that these manufacturers have offered illegal financial incentives to orthopedists, which goes against the federal Health Care Fraud and Abuse Anti-Kickback Statute for programs such as Medicare and Medicaid and may interfere in physician judgment, leading to potential harm for the patient^(5)^.

Independently of causes that generate divergence concerning therapeutic management as described in the Viola et al.^(1)^ study, actions must be taken to reduce risks to patients. Additional investigations of high methodologic rigor to develop guidelines, as well as policies to avoid conflicts of interest among physicians, pharmaceutical companies, and medical device manufacturers, constitute important actions to achieve this goal.

With this perspective, we need to reinforce that the types of medical instruments, quality of materials used in medical devices, and treatment costs are important factors; however, the focus of discussion must be the health and safety of the patient.
